# Endovascular magnetic filters for removing excess chemotherapeutic drugs from blood flow using dimensionless scaling laws

**DOI:** 10.1002/btm2.70151

**Published:** 2026-10-07

**Authors:** Won‐Seok Heo, Steven W. Hetts, Vitaliy L. Rayz

**Affiliations:** ^1^ School of Mechanical Engineering Purdue University West Lafayette Indiana USA; ^2^ Department of Radiology and Biomedical Imaging University of California San Francisco California USA; ^3^ Weldon School of Biomedical Engineering Purdue University West Lafayette Indiana USA; ^4^ Present address: Koch Institute for Integrative Cancer Research, Massachusetts Institute of Technology Cambridge Massachusetts USA

**Keywords:** dimensional analysis, hemodynamics, intra‐arterial chemotherapy, magnetophoresis

## Abstract

Intra‐arterial chemotherapy (IAC) for hepatocellular carcinoma (HCC) is limited by the escape of chemotherapeutic agents into systemic circulation, causing severe side effects such as heart failure. This study aims to develop and optimize endovascular magnetic filtration devices to enhance IAC efficacy, by targeted removal of excess drug‐coated ultrasmall superparamagnetic iron oxide (USPIO) particles from circulation. Device performance was evaluated through multi‐physics simulations that coupled the transport of drug‐coated magnetic particles in blood flow with magnetic interactions and magnetophoretic forces. A dimensional analysis framework was applied to characterize particle capture efficiency (CE) across alternative device designs, and numerical predictions were compared with published benchtop experiments. Optimized multi‐stage filters achieved up to ~80% predicted capture efficiency, while reducing the risk of intravascular device collapse. These results demonstrate a novel strategy for reducing systemic toxicity in IAC through endovascular magnetic filtration, indicating its potential to improve the safety and effectiveness of HCC treatment.

## Graphical abstract

We developed an endovascular magnetic filtration system to remove excess drug‐loaded nanoparticles during intra‐arterial chemotherapy before they spread through the body. Using dimensional analysis and multi‐physics modeling, we optimized magnetic capture efficiency while maintaining catheter safety, offering a strategy to reduce systemic toxicity and enhance targeted cancer therapy.

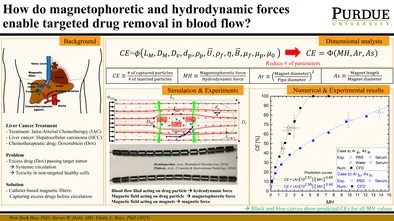

**Translational Impact Statement**

Regional chemotherapy can deliver high drug doses to tumors, but systemic leakage causes toxicity and limits efficacy. We improve a magnetic filtration approach that can be placed inside blood vessels to selectively remove excess drug‐bound nanoparticles during treatment. Using simulations and experiments with dimensionless scaling laws, we identify key biophysical design rules for clinical device performance. This work provides a mechanistic foundation for a catheter‐based filtration device with the potential to reduce off‐target toxicity and improve the safety of regional cancer therapy.

## 1 INTRODUCTION

Hepatocellular carcinoma (HCC) is the third most common cause of cancer‐related deaths in developed nations and accounts for 80–90% of primary liver cancer cases.^1^ While liver transplantation is the optimal approach for HCC treatment,^2^, ^3^ patients with unresectable tumors are treated with the intra‐arterial chemotherapy (IAC).^4^, ^5^, ^6^ In the hepatic IAC procedure, chemotherapeutic agents, such as doxorubicin (Dox), are administered via a catheter navigated to an artery supplying the tumor.^7^, ^8^, ^9^, ^10^ However, a major limitation of IAC treatment for HCC is that 50–75% of the drugs targeting the tumors instead pass into the systemic circulation via the hepatic veins beyond the tumor capillary bed. This can cause severe side effects, including irreversible heart failure, as non‐targeted healthy organs are damaged due to accumulated toxicity. This greatly limits the dosage of IAC drugs that can be safely delivered to HCC patients.^6^, ^11^, ^12^

To improve local drug retention and reduce systemic exposure, transarterial chemoembolization (TACE) was developed, combining intra‐arterial drug delivery with arterial embolization. TACE is widely used for HCC because it enables high local drug delivery, but its effectiveness is often limited by heterogeneous drug distribution and leakage of embolic agent and chemotherapeutic agents into the systemic circulation. To mitigate drug leakage, recent studies have explored advanced embolic microspheres, carrier‐based and carrier‐free delivery systems, and structurally engineered interventional materials—including multifunctional embolics and Pickering emulsions—aimed at improving intratumoral drug retention, controlled release, arterial confinement, and delivery precision.^13^, ^14^, ^15^ While these strategies primarily focus on optimizing arterial delivery and tumor retention, drug escape into the venous circulation remains an unresolved challenge. The present study addresses this limitation by introducing an endovascular magnetic filtration strategy to selectively capture excess chemotherapeutic agents downstream of intra‐arterial administration.

Endovascular filtration devices capable of removing excessive Dox doses from the bloodstream before they spread through the body have been proposed by the Chemofilter Consortium.^12^, ^16^, ^17^, ^18^, ^19^, ^20^, ^21^, ^22^, ^23^, ^24^ As shown in Figure 1a, these devices are deployed during the IAC procedure via a catheter placed in the hepatic veins and inferior vena cava (IVC) draining the liver. After the chemotherapeutics administered through the hepatic artery have affected the tumor, the device captures the excess drugs and is then retrieved via a jugular vein sheath to prevent re‐elution. Hemodynamic studies ensure minimal obstruction to blood flow while maximizing device contact area to enhance drug capture efficiency.^19^, ^24^

**FIGURE 1:**
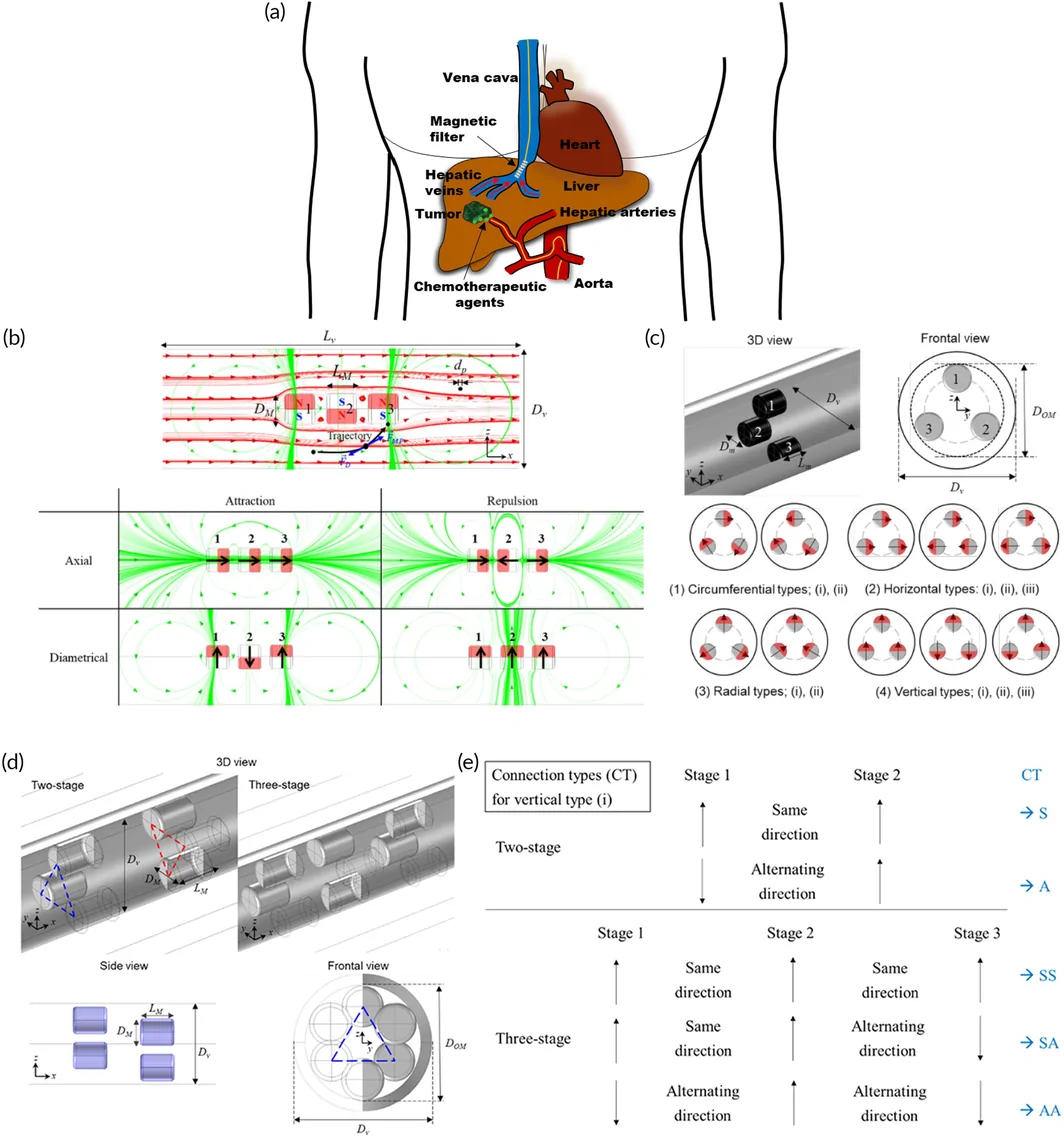
Schematics of the magnetic filtration device placed in the vein draining the tumor to capture chemotherapeutic drugs bound to the ultrasmall superparamagnetic iron oxide (USPIO) particles. (a) Human anatomical diagram. (b) Series filters: Three cylindrical magnets deployed in series along the centerline of a blood vessel. Four models with different magnetic fields resulting from alternative magnetic force directions between the magnets (attraction vs. repulsion) and two types of the magnetization direction in the cylindrical magnets (axial vs. diametrical). The black dots represent magnetic particles suspended in the blood. The red lines are the flow streamlines around the magnets. The green lines are tangent to the magnetic flux density, $\overset{⃑}{B}$, generated by the three magnets. (c) Parallel (single‐stage) filters: Three cylindrical magnets with diametrical magnetization type deployed in parallel. Ten models with different magnetic fields resulting from the four configurations: (1) Circumferential, (2) Horizontal, (3) Radial, and (4) Vertical configurations. The black arrows depict the directions of magnetization. (d) Multi‐stage filters: Several stages placed in a staggered arrangement. (e) Example of the multi‐stage connection types for vertical type (i) where magnetizations of the three magnets for a single stage are unidirectional. Two‐stage filters have two different device configurations, while three‐stage filters have three different device configurations.

Alternative drug‐binding methodologies for endovascular filters include genomic DNA‐Dox binding,^12^ electrostatic attraction to anionic polystyrene sulfonated ion‐exchange resins for Dox affinity,^16^, ^17^, ^18^, ^19^ and magnetic attraction.^20^, ^21^, ^22^, ^23^ In the genomic DNA approach, chemotherapeutic drugs are bound through DNA base intercalation.^12^ For the ion‐exchange methods, positively charged Dox in the blood is attracted to negatively charged ion‐exchange resins containing a strong acid sulfonate group on the device's surface.^16^, ^17^ In vitro experiments with ion‐exchange filters incorporating block copolymer membranes demonstrated 90% Dox removal within 31 min from phosphate‐buffered saline (PBS) at rest,^18^ but this approach was limited by the mechanical fragility of the membranes. Alternatively, experimental studies with durable magnetic filters have shown effective capture of chemotherapeutic‐coated magnetic particles using applied magnetic fields.^20^, ^21^, ^22^, ^23^ These alternative filtration methods show promise in reducing systemic toxicity in preclinical and in vitro studies.

Magnetic filtration is an attractive method for drug capture since magnetic fields act over relatively wide ranges without affecting biological tissues. Superparamagnetic iron oxide nanoparticles (SPIONs) such as maghemite (gamma ferric oxide, γFe_2_O_3_) and magnetite (ferric oxide or lodestone, Fe_3_O_4_) are well‐suited for clinical applications since their magnetic behavior only occurs when exposed to a magnetic field. Superparamagnets are typically made of iron oxide or other ferrous materials, with extremely small sizes (10–100 nm).^25^ These properties of SPIONs make them ideal for applications such as hyperthermia, biomagnetic separation, magnetic drug targeting, and imaging.^26^ Magnetic filters can be designed to adsorb the Dox bound to the Ferumoxytol (Feraheme) which is one of the types of magnetic iron oxide nanoparticles (IONPs) approved by the US Food and Drug Administration (FDA). Modeling studies have shown the simulated capture of SPION‐related materials onto small ferromagnetic wires and magnets in good agreement with experiments, indicating the feasibility of magnetic filters.^21^, ^22^, ^23^, ^27^, ^28^, ^29^, ^30^, ^31^, ^32^, ^33^, ^34^ Other computational works have employed force‐based Eulerian–Lagrangian frameworks to examine magnetic particle targeting in arterial and cerebrovascular systems, including patient‐specific geometries and pulsatile flow, showing that flow unsteadiness primarily modulates targeting efficiency without altering the underlying magnetophoretic transport physics.^35^, ^36^, ^37^, ^38^

In this study, we modeled alternative magnetic filter configurations and assessed the magnetophoretic mobility of SPIONs to predict their transport and capture. We identified the optimal device design for maximizing particle capture using Eulerian analyses of the hemodynamic and magnetic fields and Lagrangian particle transport. Computational results were compared with in vitro data^22^, ^23^ and predictive performance curves derived from dimensionless numbers developed in our prior work.^39^ Unlike formulation‐specific magnetic drug delivery studies, this work focused on scaling‐based capture behavior across clinically relevant particle sizes spanning the nano‐to‐micro range, enabling identification of optimal particle sizes that balance magnetophoretic control and embolic safety constraints. We further investigated the inter‐magnet interactions using the Maxwell stress tensor to assess device stability and evaluated magnetophoretic forces between particles and magnets. This computational framework provides a predictive methodology for designing endovascular magnetic filters, enabling evaluation of alternative concepts and configurations while reducing the number of required animal studies.

## 2 METHODS

In this study, no ethical considerations are required because no animal or human tests were conducted.

### 2.1 Magnetic filtration device configurations

The magnetic filtration device comprising cylindrical magnets (SuperMagnetMan Magnetics, Pelham, AL) was placed in an idealized blood vessel where the magnetic particles were suspended in blood. The magnets could produce magnetic fields sufficiently strong to saturate the magnetization of Dox (~1.5 nm)‐loaded magnetic particles ranging from 10‐nm to 100‐μm sizes so that the particles behaved as permanent magnets. Alternative magnet configurations that might reduce the hydrodynamic drag and increase the magnetophoretic force were considered in order to optimize the performance of the magnetic filters. We modeled series, parallel (single‐stage), and multi‐stage filters as shown in Figure 1b–e. The number of magnets remained the same for both series and parallel filters and increased for multi‐stage filters.

Figure 1b depicts the filters with magnets deployed in series along the vessel's center. There are two types of cylindrical magnets based on their magnetization direction. A magnet with magnetization along its axis has two poles at the ends, while magnetization across the magnet diameter results in two poles located at each half‐circle surface, as shown in Figure 1b. Two adjacent magnets in series filters were set to have different magnetic force directions. Attraction forces occurred when the magnets were arranged with opposite polarities facing each other, while repulsion forces occurred when adjacent faces had the same polarity. Four models resulted from different magnetic force directions between the magnets (attraction vs. repulsion) and two types of magnetization directions in the cylindrical magnets (axial vs. diametrical). The center of the 2nd magnet was placed at the midpoint ($L_{v}/2$) of the model's *x*‐coordinate, and the magnets were placed 1 mm apart from each other. The computational models herein were compared with the previously reported experimental results.^23^

Figure 1c shows the filters with magnets deployed in parallel near the vessel wall. The three magnets are positioned circumferentially 120 degrees apart from each other within the outermost circle diameter, $D_{\mathrm{OM}}$, equal to 0.8 of the vessel diameter. Ten alternative filter configurations resulted from four types of arrangements (circumferential, horizontal, radial, and vertical types) for diametrically magnetized magnets. In this work, these parallel filters are considered single‐stage filters.^40^

The multi‐stage filters consisted of several stages where the magnets of neighboring stages were placed in a staggered arrangement, shifting their angles by 180 degrees for flow mixing (Figure 1d). The number of magnets, N, was three per stage, with up to four stages, m. The distance between stages was equal to the length of a single magnet. When adding a new stage downstream, the magnetization direction can either align with or alternate from the upstream stage, introducing connection types (CT) of the same or alternating direction. For instance, Figure 1e illustrates multi‐stage filters of vertical type (i) with different CTs. Similarly, a two‐stage filter of circumferential type (i) with the same CT demonstrates clockwise or counter‐clockwise magnetization, while the alternating CT results in clockwise to counter‐clockwise magnetization or vice versa. In the case of horizontal type (i) with the alternating CT, horizontally opposing magnetization directions between adjacent stages are considered. For radial type (i) with the same CT, magnets are directed inward or outward, whereas the alternating CT results in out‐in or in‐out magnetization directions between stages.

### 2.2 Magnetophoretic scaling arguments determining the particle CE

The particle capture efficiency (CE) was calculated for individual magnets and for each stage in multi‐stage filters. The particle CE is defined as the fraction of injected particles captured by the filter:

(1) $$\mathrm{CE}=N_{c}/N_{i}$$

where $N_{c}$ and $N_{i}$ are the number of captured particles and the number of injected particles through the blood vessel, respectively.

The particle CE (Equation 1) depends on the geometrical characteristics, including the number of magnets, N, number of stages, m, length and diameter of the magnet, diameters of the fluid vessel and particle ($L_{M}$, $D_{M}$, $D_{v}$, and $d_{p}$), fluid velocity relative to the particle, $\overset{⃑}{U}$, fluid dynamic viscosity, η, particle and fluid densities ($\rho_{p}$ and $\rho_{f}$). For the magnetic field, relevant variables are the magnetic flux density, $\overset{⃑}{B}$, and magnetic permeabilities of the particle, fluid, and free space ($\mu_{p}$, $\mu_{f}$, and $\mu_{0}$). These 15 relevant variables can be combined to 11 scaling arguments through dimensional analysis (all variables are listed in Table S1):^40^

(2) CE=Φμr,f,KMP,MH,Ar,IH,As,ρp/ρf,Rev,N,m

where $\mu_{r,f}=\frac{\mu_{f}}{\mu_{0}}$ is the relative magnetic permeability of the fluid, $K_{\mathrm{MP}}=\frac{\mu_{p}-\mu_{f}}{\mu_{p}+2\mu_{f}}$ is the magnetic Clausius‐Mossotti factor, $\mathrm{MH}=K_{\mathrm{MP}}\frac{B^{2}{d_{p}}^{2}}{\eta \mu_{f}UD_{v}}$ is the ratio of the magnetophoretic force to hydrodynamic drag which is proportional to the fluid magnetic energy density, $\frac{B^{2}}{2\mu_{f}}$, and $\mathrm{IH}=\frac{\rho_{p}U{d_{p}}^{2}}{\eta L_{M}}$ is the ratio of the particle inertial force to hydrodynamic drag.^39^ Herein, $\mathrm{Ar}=\frac{{D_{M}}^{2}}{{D_{v}}^{2}}$ is the magnet to vessel cross‐sectional area ratio which is closely related to the distance between the magnet and particle, assuming the particles are uniformly distributed throughout the vessel's cross‐sectional area, $\mathrm{As}=\frac{L_{M}}{D_{M}}$ is the magnet aspect ratio, and Rev=ρfUDvη is the vessel Reynolds number. These 10 independent parameters determine the particle CE describing the magnetophoretic phenomena around magnetic filters in the blood vessel.

In this study, the functional relationship determining the particle CE (2) can be simplified by considering the relative importance of each dimensionless group (See details in Supporting Information S4). The relative magnetic permeability of blood, $\mu_{r,b}$, and magnetic Clusius‐Mossotti factor, $K_{\mathrm{MP}}$, are assumed 1. Thus, the two unitless parameters considered as a constant of unity can be ignored from the functional relationship (2). In addition, the previously reported experimental and computational results revealed that the particle CE is not affected by the dimensionless numbers of the density ratio, $\rho_{p}/\rho_{f}$, and vessel Reynolds number, Rev, when keeping the other dimensionless numbers in (2) fixed. Lastly, IH has a negligible effect on the particle CE when other dimensionless numbers are held constant. Hence, the number of the independent dimensionless numbers determining the particle CE in (Equation 4) can be reduced from 10 to 5 by ignoring the relative magnetic permeability of the fluid, $\mu_{r,f}$, magnetic Clausius‐Mossotti factor, $K_{\mathrm{MP}}$, particle inertial to hydrodynamic force ratio, IH, density ratio, $\rho_{p}/\rho_{f}$, and vessel Reynolds number, Rev.^39^

### 2.3 Numerical implementation

The Eulerian–Lagrangian method was used to solve particle motion in blood flow using a finite element solver, COMSOL Multiphysics (Stockholm, Sweden), with the Laminar flow, AC/DC, and Particle Tracing modules. The hemodynamic and magnetic fields were computed with the Eulerian approach. Subsequently, particle motion was simulated in the Lagrangian frame of reference. All governing equations describing blood flow and magnetic fields as well as particle motion were computed in the same set of mesh elements. Numerical analysis focused on optimizing the capture of magnetic particles characterized with dimensionless numbers (See details in Supporting Information S1 and S2).

Blood flow in the hepatic vein (Equations S2 and S3) was computed using the dynamic viscosity, η, of 0.00345 Pa·s and density, $\rho_{f}$, of 1060 kg/m^3^. The hepatic vein with a diameter of 1 cm was encompassed by a surrounding tissue to obtain a continuous magnetic field inside and outside the vessel.^41^ To facilitate comparison with the previously published experiments^22^, ^23^ and simulation results,^39^ the magnet with dimensions ($L_{M}=D_{M}=2.5$ mm) and a fillet radius (0.25 mm) was modeled. This configuration resulted in the magnet to vessel cross‐section area ratio, Ar, of 0.0625. The average flow velocity, ${\bar{u}}_{f}$, was 5 cm/s based on blood flow in the hepatic vein.^42^ Since the human hepatic vein length is approximately 2 cm, the filter was placed within 2 cm of the modeled vessel length, $L_{v}$, of 10 cm.^43^ The vessel wall was modeled as a rigid tube because wall compliance was considered to have a secondary effect on the filter performance. A parabolic velocity profile was set up at the vessel inlet, while constant pressure was set at the outlet. For the vessel wall and surfaces of the filter, no‐slip conditions were applied. Magnetic fields (Equations S5–S7) were computed for all domains treated as magnetic materials including surrounding tissues (extravascular space), vessel wall, blood, magnets, and particles. The cylindrical magnet was modeled to create a constant magnetic field with the magnetic remanent flux density of the magnet, ${\overset{⃑}{B}}_{r,M}$, of 1.45 T. Since the magnetic susceptibilities of the surrounding tissue and blood are nearly zero, the relative magnetic permeabilities of the tissue and blood, $\mu_{r,t}$ and $\mu_{r,b}$, were set to 1. The magnetic insulating boundary condition was applied to the surface of the surrounding tissue ($\overset{⃑}{B}\cdot \hat{n}=0$). In the Lagrangian approach, the time marching solver was used for the trajectories of the magnetic particles (Equation S1) based on the obtained numerical solutions of both flow and magnetic fields. The “one‐way coupling” approach was employed since the impact of particle movement on magnetic and flow fields can be ignored for a low concentration of particles in the flow.^39^ In this study, the baseline particle diameter was set to 1 μm, according to the previously reported experimental data^22^, ^23^ and simulations.^39^ The relative magnetic permeability of the particle, $\mu_{r,p}$, of 4000 and density, $\rho_{p}$, of 5240 kg/m^3^ were specified for the particles. Five hundred magnetic particles were injected at the inlet of the model with 0.2 s interval, and the injection continued for 0.4 s, resulting in 1500 particles total, $N_{i}$. The particles were assumed to be uniformly distributed at the vessel inlet. The outlet surface was set to allow particles to escape. The particles were allowed to bounce or reflect from the rigid vessel wall, while the surfaces of the filters were set to trap particles for calculating the particle CE (Equation 1). Numerical simulations were conducted for a range of input parameters to evaluate the dimensionless groups derived above and determine their effect on the particle CE. The parameters related to the hepatic vein geometry and blood characteristics remained the same, while other input parameters varied maintaining the same dimensionless numbers. Thus, particle sizes ranging from half to twice the baseline value of 1 μm were tested, along with flow velocities varying from one‐quarter to twice the baseline value of 5 cm/s.

## 3 RESULTS

The magnetic filtration devices modeled in the study consist of cylindrical magnets (SuperMagnetMan Magnetics, Pelham, AL) deployed in an idealized blood vessel with magnetic particles suspended in blood flow. The magnets produced magnetic fields sufficient to magnetize Dox‐loaded particles (10‐nm to 100‐μm), causing them to behave as permanent magnets. This study compared series, parallel (single‐stage), and multi‐stage filter configurations (Figure 1b–e), with three magnets in both series and parallel (single‐stage) filters and additional magnets for multi‐stage filters (see Section 2). The performance of these devices was evaluated based on computational results and compared with the previously reported experiments.^22^, ^23^ The dynamic behavior of particles within the filters was found to be primarily defined by the interplay between magnetophoretic force and Stokes' viscous drag (Equation S1). Various magnet configurations were explored to reduce hydrodynamic drag and increase magnetophoretic force, thereby optimizing the performance of the filtration devices. Below we present computational results obtained for each filter configuration, characterizing device's performance and considering magnetic forces between the magnets. The theoretical backgrounds for magnetic particle transport inside vessels are provided in Supporting Information S1.1.

### 3.1 Particle CEs and magnetic forces for various filter configurations

Series filters, as shown in Figure 1b, can have four different configurations based on the orientations of the magnetic force and magnet's magnetization (Section 2). These were classified into two types by overall filter magnetization direction: unidirectional magnetization indicates filters having attraction forces with axially magnetized magnets or repulsion forces with diametrically magnetized magnets. In contrast, alternating magnetization indicates filters having attraction forces with diametrically magnetized magnets or repulsion forces with axially magnetized magnets. Filters with alternating magnetization demonstrated higher CEs than those with unidirectional magnetization, where CE increased by approximately 15% from 5.2% (axial) to 6.0% (diametrical) (Table 1). Experimental results corroborated these findings, showing 14% and 17% increases in CE for diametrical filters in PBS and porcine serum, respectively.^23^ Magnetic forces between the magnets in series filters were not considered since rubber spacers between the individual magnets prevented magnetic filter collapse.^22^, ^23^

**TABLE 1:** Particle capture efficiencies for the filters deployed in series.

Particle CE [%]		Magnetic force direction	
		Attraction	Repulsion
Magnetized direction	Axial	3.2	5.2
	Diametrical	6.0	4.8

Parallel (single‐stage) filters consisted of ten models classified into four magnetization types, where each type has two or three different configurations (Section 2). In type (i), magnets exhibit unidirectional magnetization in the circumferential, horizontal, radial, and vertical direction, respectively, whereas types (ii) and (iii) lack any unidirectional magnetization (Figure 1c). As shown in Table 2, types (ii) and (iii) generally achieved higher or equal CEs compared to each unidirectional type (i), except for circumferential types. The CEs for horizontal and vertical types (iii) were superior for each comparison. Parallel filters achieved CEs approximately 2.7 times higher than series filters. Table 2 provides the resultant maximum magnetic forces exerted on magnets using the Maxwell magnetic stress tensor (Supporting Information S3). Among the different filter models, radial type (ii) exhibited the strongest force at 313 mN, indicating significant magnetic interactions. In contrast, horizontal type (i) and vertical type (i) displayed relatively weaker forces of 110 mN and 130 mN, respectively. Notably, all models' net magnetic forces are zero, while horizontal and vertical types (iii) exhibit non‐zero net moments leading to rotational force (Figures S3 and S5). Consequently, horizontal and vertical types (iii) were excluded from the possible filter designs despite their high capture performance. Instead, horizontal and vertical type (i) configurations were further considered as potential design options due to their weak interactions.

**TABLE 2:** Particle capture efficiencies and the maximum magnetic forces for the parallel (single‐stage) and multi‐stage filters.

ParticleCE [%], Magnetic force$\;F_{m}$ [mN]			Arrangement type			
			Circumferential	Horizontal	Radial	Vertical
Alternative models for parallel (1‐stage) filter	(i)	CE [%]	13.2	12.0	12.4	13.2
		$F_{m}$ [mN]	230	110	308	130
	(ii)	CE [%]	12.8	12.4	12.4	13.2
		$F_{m}$ [mN]	220	299	313	250
	(iii)	CE [%]	—	13.2	—	14.4
		$F_{m}$ [mN]	—	295	—	280

2‐stage	Horizontal (i)		Radial (i)		Vertical (i)	
CT	Same	Alternating	Same	Alternating	Same	Alternating
CE [%]	26.8	26.5	25.9	25.6	27.6	27.6
$F_{m}$ [mN]	52	176	313	303	53	205

3‐stage	Horizontal (i)			Vertical (i)		
CT	SS	SA	AA	SS	SA	AA
CE [%]	32.4	33.2	33.2	34.8	34.6	34.3
$F_{m}$ [mN]	46	182	239	46	212	281

4‐stage	Horizontal (i)			Vertical (i)		
CT	SSS	SAS	ASA	SSS	SAS	ASA
CE [%]	40.4	40.7	41.6	41.2	39.9	40.0
$F_{m}$ [mN]	44	120	181	45	136	210

Multi‐stage filters were created by aligning parallel filters in a row (Figure 1d,e) up to four stages with two different (same, S, and alternating, A) connection types (CTs, see Section 2). Table 2 summarizes the performance of multi‐stage filters based on the number of stages. For two‐stage filters, the circumferential type (i) filter was excluded due to middle‐range magnetic force (230 mN) with similar particle CE compared to other single‐stage filters. While no significant differences in CE were observed among radial, horizontal, and vertical types as well as between CTs, substantial variations in maximum magnetic forces were noted. Horizontal and vertical type (i) filters with the same CT demonstrated significantly weaker magnetic forces than those with alternating CT, making them structurally advantageous for preventing magnet collapse in blood vessels. These properties suggest potential for further exploration in three‐stage filter designs. For three‐stage filters, magnetic forces decreased with additional stages connected with the sequentially same connection type (SS), while the sequentially alternating (AA) connection types showed increases in the forces in each case. Magnetic forces for SA types of three‐stage filters were similar to alternating CTs of two‐stage filters. Although magnetic forces varied from 46 mN to 281 mN, particle CEs stayed similar at 33–34.5%. The SS and SA types, thus, were further studied to identify the lowest magnetic forces with higher CE. In four‐stage filters, the average CEs across the three CTs were approximately 41% for horizontal type (i) and 40% for vertical type (i). Magnetic forces of SSS types (4‐stage) slightly decreased from SS types (3‐stage). ASA types of four‐stage filters showed similar forces to SA types of three‐stage filters and alternating CTs of two‐stage filters with approximately 180 mN and 210 mN, respectively. Across all multi‐stage filters, CEs increased as a new stage was added, while the maximum magnetic forces decreased with the same CT and increased with the alternating CT. Connecting stages with the same direction offset magnetic interactions between magnets, reducing the forces on filters. With the same CT, particle CEs for vertical type (i) were higher than those for horizontal type (i) across stages. Consequently, the prospective design configurations were narrowed down to vertical type (i) filters with the same CTs.

Vertical type (i) showed higher CEs with moderate magnetic forces, indicating no relationship between the CE and magnetic force. To understand the relationship between the magnetic force and CE, the Maxwell magnetic stress tensor, ${\overset{̿}{T}}_{m}$, and magnetic flux density, $\overset{⃑}{B}$, were analyzed. Figure 2a shows the distribution of the Maxwell magnetic stress tensor norm on the force probe surface (Supporting Information S2.1) for three‐stage vertical type (i) with SS and AA connection types. Despite a similar CE of approximately 34.5%, the AA type exhibited a magnetic force six times larger (281 mN) than the SS type (46 mN). The substantial difference in the forces can be qualitatively reflected in the norm of the stress tensor over the force probe surface (Supporting Information S1.2 and S2.1). Figure 2a shows higher stress tensor values depicted in red, spread between magnets, for the AA type (approximately 4.5 × 10^4^ N/m^2^) compared to the SS type (approximately 3.25 × 10^4^ N/m^2^). Despite different magnetic forces, both SS and AA types exhibited almost identical magnitude of magnetic flux density $\left\|\overset{⃑}{B}\right\|$ and CE (Figure 2b). This suggests that the $\overset{⃑}{B}$ fields themselves influence CEs rather than magnetic forces. A qualitative comparison is possible using the distribution of the Maxwell stress tensor without calculating the exact direction and magnitude of the magnetic force. The CE is not directly related to magnet‐magnet forces; rather, reducing inter‐magnet interactions improves structural stability without degrading particle capture, which is controlled by local magnetic field gradients on individual particles.

**FIGURE 2:**
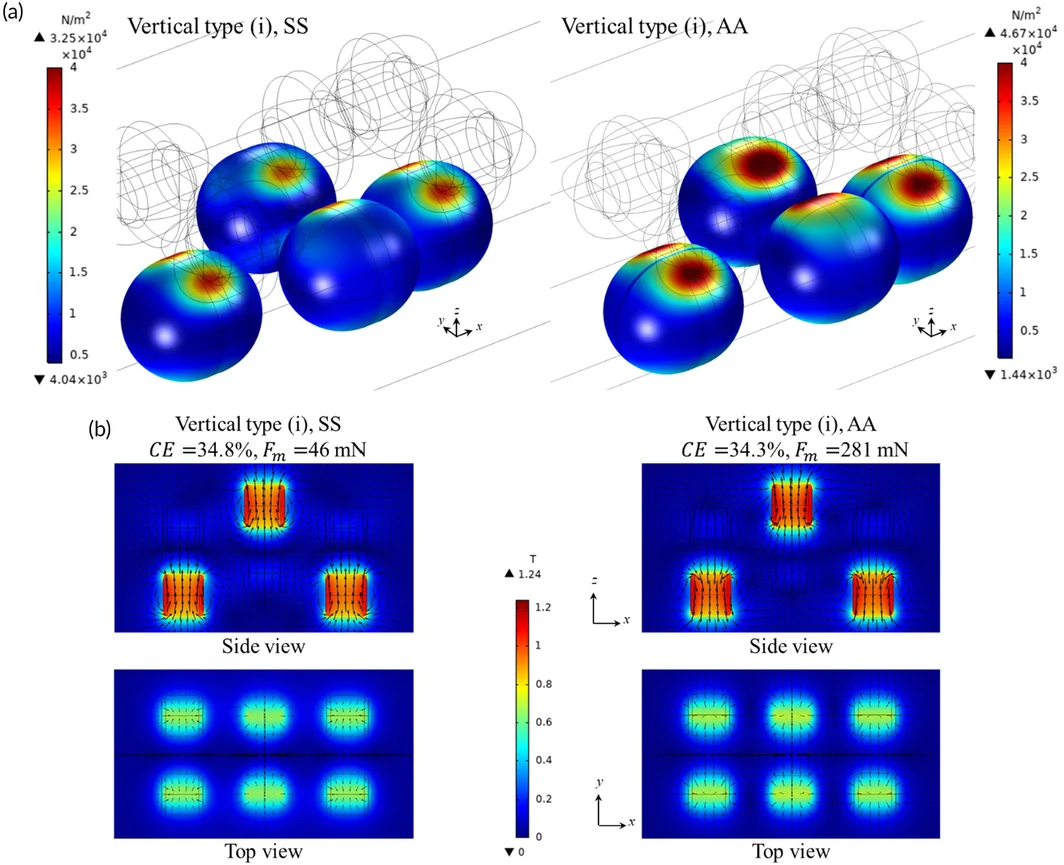
Maxwell magnetic stress tensor and magnetic flux density for three‐stage filters of vertical types (i) with the SS and AA connection types. (a) Contour plots of the Maxwell magnetic stress tensor norm, $\left\|{\overset{̿}{T}}_{m}\right\|$. (b) The magnitude distribution of magnetic flux density, $\overset{⃑}{B}$.

### 3.2 Particle CEs for each magnet/stage along the blood vessel

Table 3 presents CEs for individual magnets in series filters and individual stages in multi‐stage filters along the blood flow direction. In the series filters, most particles were captured by the upstream magnets for both axial (2.4%) and diametrical (2.8%) configurations, with CEs decreasing downstream due to fewer available particles for capture along the filter's length. In unidirectional magnetization filters (Axial‐Attraction and Diametrical‐Repulsion), particle accumulation occurred mainly on the first or the first two magnets, while the CE on the third magnet was relatively low at 0.8% for both. This pattern suggests that unidirectional filters, acting like a single elongated magnet, lacked magnetic field interaction, limiting particle attraction to the third magnet. Conversely, alternating filters (Axial‐Repulsion and Diametrical‐Attraction) exhibited higher CE on the third magnets (2.0% and 2.4%), indicating that particles mainly accumulated on both ends of the three magnets. This distribution reflects sufficient magnetic field interaction, enhancing particle capture across the filter. For individual stages of vertical type (i) in multi‐stage filters, CEs ranged from 11.6% to 14.0% for the first two upstream stages and dropped to 7.4–9.2% for the last two stages. Particles flowing through the magnets of the 1st stage encountered the magnets of the 2nd stage, which was staggered by a 180‐degree rotation on the y‐z plane, referring to the red triangle (Figure 1d). After passing the 2nd stage, the number of available particles for capture significantly decreased along the vessel. In terms of each stage's performance, considered as the fraction of passing particles through the upstream stage captured by the downstream stage, each stage CE exceeds 10.2% and maintains an average of 13.2%.

**TABLE 3:** Particle capture efficiency, CE, for each magnet in series filters and for each stage in multi‐stage filters.

CE for series filters		1st magnet	2nd magnet	3rd magnet	Total
Magnetization	Magnetic force	%	%	%	%
Axial	Attraction	2.4	0	0.8	3.2
	Repulsion	2.4	0.8	2.0	5.2
Diametrical	Attraction	2.8	0.8	2.4	6.0
	Repulsion	2.8	1.2	0.8	4.8

CE for multi‐stage filters		1st stage	2nd stage	3rd stage	4th stage	Total
# of stages, m	Vertical (i)'s CT	%	%	%	%	%
1	—	13.2	—	—	—	13.2
2	S (Same)	13.6	14.0	—	—	27.6
3	SS	13.6	13.8	7.4	—	34.8
4	SSS	12.8	11.6	9.2	7.6	41.2

### 3.3 Effects of hemodynamic and magnetic fields on series and parallel filters

As shown in Figure 3, blood velocity fields were computed for the series and parallel filters. The maximum blood speed for the series filter was approximately 10.3 cm/s, and 12.4 cm/s for the parallel filter (Figure 3a). The recirculating flow with closed streamlines was observed downstream of the filters. In Figure 3b, the flow fields measured on cross‐section plane A‐A were expressed as the average blood speed at a radius relative to the mean hepatic vein blood speed plotted against the distance from the centerline of the vessel normalized by the hepatic vein radius. For the series filter, the maximum speed on the cross‐section plane was observed approximately 1.8 times higher than the mean speed of the hepatic vein flow ($r/R_{v}\approx 0.5$), while for the parallel filter, it occurred along the centerline of the vessel ($r/R_{v}=0$) at approximately 2.3 times higher than the mean speed. Figure 3c,d show the magnetic fields generated by the filters which affect the magnetophoretic force exerted on the particles. In the series filters, one filter exhibits unidirectional magnetization with the axial‐attraction type, while the other exhibits alternating magnetization with the diametrical‐attraction type. In the side view figure, the magnetic flux density for both filters ranges from 0 to approximately 1.2 T (Figure 3c). The unidirectional filter showed denser magnetic fields in the axial direction while the alternating filter showed wider fields in the radial direction. The parallel filter consisting of diametrically magnetized magnets showed the magnetic flux density ranging from 0 to approximately 0.55 T in the side view figure. However, the magnetic fields measured on cross‐section plane A‐A represented different maximum magnetic flux densities of 1.2, 1, and 0.9 T in order, respectively. Although the axially magnetized magnets deployed in series showed the highest magnetic flux density of 1.2 T, the diametrically magnetized magnets deployed in parallel created a wider region of the magnetic field throughout the cross‐section of the vessel. As shown in Figure 3d, the magnitude of the magnetic flux density was inversely proportional to the distance away from the magnet, where the distance was normalized by the vessel radius. For the series filters, the magnetic flux density for the axially magnetized magnets (black curve) was approximately 0.17 T at a normalized radius of 0.25 and decayed below 0.1 T beyond the radius of ~0.35. For diametrically magnetized magnets (blue curve), the magnetic flux density was about 0.75 T at the radius of 0.25 and dropped below 0.1 T beyond the radius of ~0.5. In contrast, the parallel filter (red curve) exhibited a magnetic flux density ranging from 0 at the center to approximately 0.4 T at the radius of 0.3, with an average value of 0.23 T across all radii.

**FIGURE 3:**
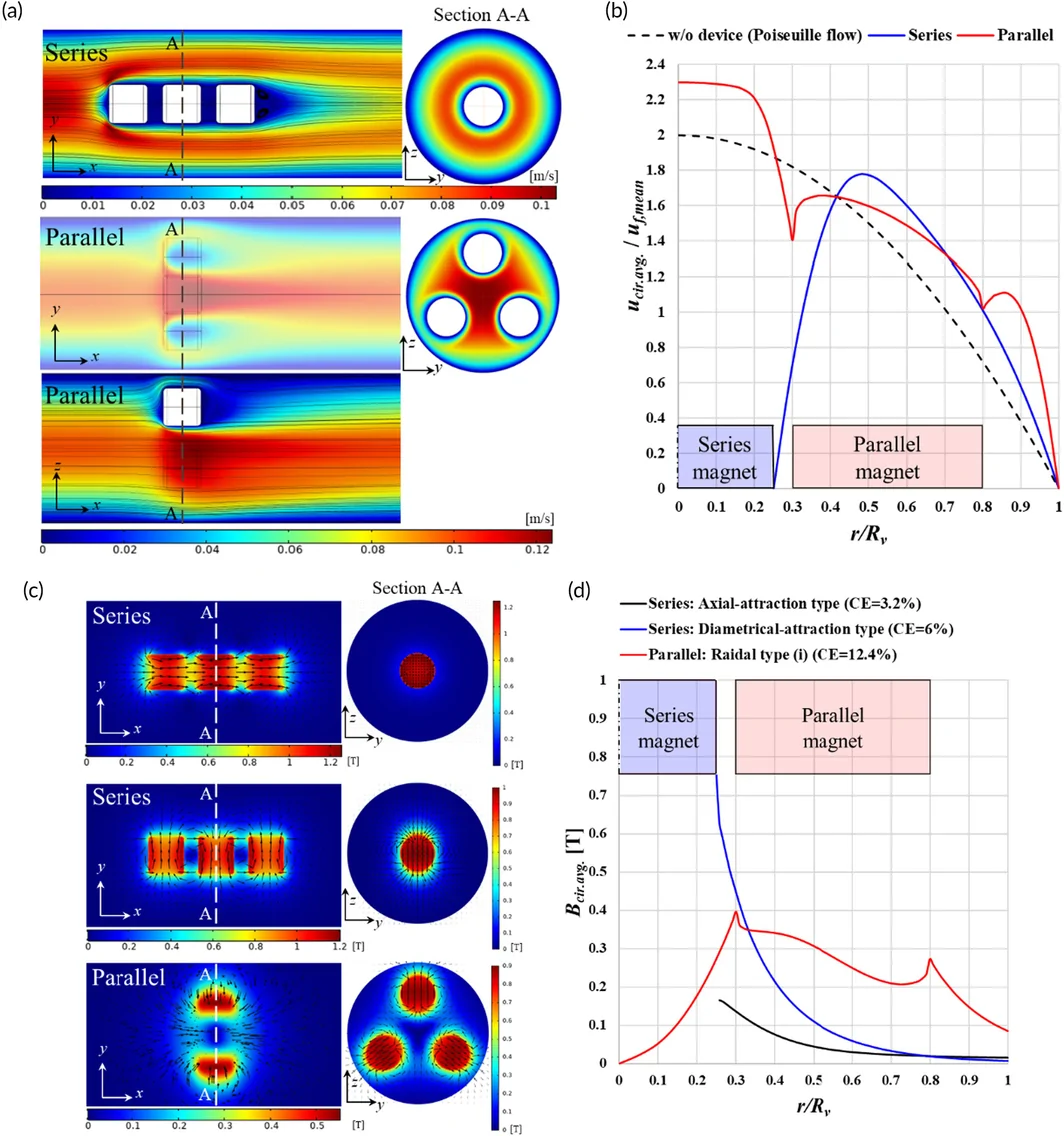
Predicted flow and magnetic fields distribution. Side and frontal views for the series and parallel deployments of the three cylindrical magnets; (a) Flow fields with streamlines and (c) Magnetic fields for Series‐Axial‐Attraction, Series‐Diametrical‐Attraction, and Parallel‐Diametrical‐Radial (i), respectively, where the black arrows depict directions of magnetic flux density. Circumferentially averaged profiles on the frontal area along cross‐plane A‐A for the series and parallel deployments of the filters; (b) Velocity profiles normalized by ${\bar{u}}_{f}$ and parabolic velocity profile for the flow with no devices in the vessel and (d) Magnetic flux density profiles. Series and parallel magnets describe the cylindrical magnets deployed in series (0 < $r/R_{v}$ < 0.25) and parallel (0.3 < $r/R_{v}$ < 0.8).

The Stokes' drag (Equation S4) depends on the blood velocity relative to the particle, while the magnetophoretic force (Equation S8) is governed by the magnitudes of magnetic flux density and its gradient, which are inversely proportional to the distance and the distance squared from the magnet, respectively.^34^ In regions far from the magnets, particle motion is mainly governed by hydrodynamic drag rather than magnetophoretic force, as particles only feel the magnetic field gradient over a short distance. For magnets deployed in series, strong magnetic fields near the vessel's center attract particles, while weaker fields near the wall limit attraction due to distance from the magnets. Along the normalized radius up to approximately 0.5, blood speed increased (blue curve in Figure 3b) as magnetic fields decreased (blue and black curves in Figure 3d), where magnetophoretic force could effectively overcome hydrodynamic drag. Generally, attraction between opposite poles generates concentrated magnetic fields, while repulsion facing the same poles spreads out field lines, resulting in a diffused (wider flux path) magnetic field. Unidirectional filters create concentrated fields, whereas alternating filters produce diffuse fields (Figure 1b). This diffused magnetic behavior slows down flow‐driven particles, leading to a higher CE for the alternating filter (6.0%) than the unidirectional filter (3.2%) as shown in Figure 3d. In a parallel deployment near the vessel wall, where velocity is significantly lower than in the core flow (Figure 3a,b), particles are more likely to be captured due to reduced hydrodynamic drag. Compared to series filters, parallel filters exhibited higher magnetic flux density across radii, leading to substantially higher CE (12.4% vs. 3.2% and 6.0%) as shown in Figure 3d. Unidirectional arrangements of horizontal and vertical types (i) such as a bulk magnet with the same magnetization direction, produced insufficient magnetic fields compared to types (ii) and (iii) (Table 2). Unlike series filters, the order of individual magnets in parallel filters is not relevant, instead, the average CE on each magnet for parallel filters (4.2%) is significantly higher than for series filters (1.6%) as shown in Table 3. Disparities in magnetic fields between series and parallel filters facilitate improved retention of magnetic particles on parallel filters. These findings highlight the ability to increase CE by adjusting magnet arrangements without adding more magnets.

### 3.4 Magnetic field and its gradient determining the magnetophoretic force

The particle CE was primarily influenced by magnetic flux density rather than magnetic forces between magnets. The magnetophoretic force directly affects the particle CE and is governed by the magnetic flux density, $\overset{⃑}{B}$, its gradient, $\nabla \overset{⃑}{B}$, and their dot product, $\overset{⃑}{B}\cdot \nabla \overset{⃑}{B}$ (Equation S8). Figure 4 illustrates how the magnetic flux density dictates the particle CE for three‐stage filters of horizontal and vertical types (i) with the same CT. In Figure 4a, where the norm of the $\overset{⃑}{B}\cdot \nabla \overset{⃑}{B}$ field ranges from 0 to 1000 T^2^/m, the fields showed a significant disparity despite identical magnetic forces of 46 mN for both designs. The vector fields for vertical type (i) were approximately two orders of magnitude higher than those for horizontal type (i), leading to a higher particle CE of 34.8% for vertical type (i) compared to 32.4% for horizontal type (i). The magnetic flux density, $\overset{⃑}{B}$, is depicted in Figure 4b, showing a quantitative difference in magnitudes between the $\overset{⃑}{B}$ fields for the two filters, with vertical type (i) having a higher magnitude. The magnetic flux density gradient, $\nabla \overset{⃑}{B}$, is shown on the x‐y plane (top view), ranging from 0 to 1000 T/m, where two vector components are displayed for both filters (Figure 4c). The values for $B_{x}$ and $\partial_{x}\overset{⃑}{B}$ were insignificantly small, thus $\nabla \overset{⃑}{B}$ fields were sufficiently represented by the other two vector fields of $\nabla B_{y}$ and $\nabla B_{z}$, respectively (Supporting Information S1.3). The $\nabla \overset{⃑}{B}$ fields for vertical type (i) were higher, depicted with the dark red regions, compared to horizontal type (i), indicating that the vertical configuration creates stronger localized magnetic gradients. This suggests that vertical type (i) is more effective in enhancing magnetophoretic forces, crucial for improving CE. This result with the $\overset{⃑}{B}$ and $\nabla \overset{⃑}{B}$ fields in Figure 4b‐c indicated that the $\overset{⃑}{B}\cdot \nabla \overset{⃑}{B}$ fields, rather than $\overset{⃑}{B}$ or $\nabla \overset{⃑}{B}$ fields, could be a more important factor in the magnetophoretic force. In this study, the magnitudes for these parameters were quantitatively plotted, leading to consistent conclusions. By calculating the spatial derivative of the magnetic fields using Lagrange elements (as detailed in Supporting Information S2.2), particle CE was qualitatively predicted without the need for additional particle motion simulations involving intensive time‐marching trajectory analysis of magnetic particles.

**FIGURE 4:**
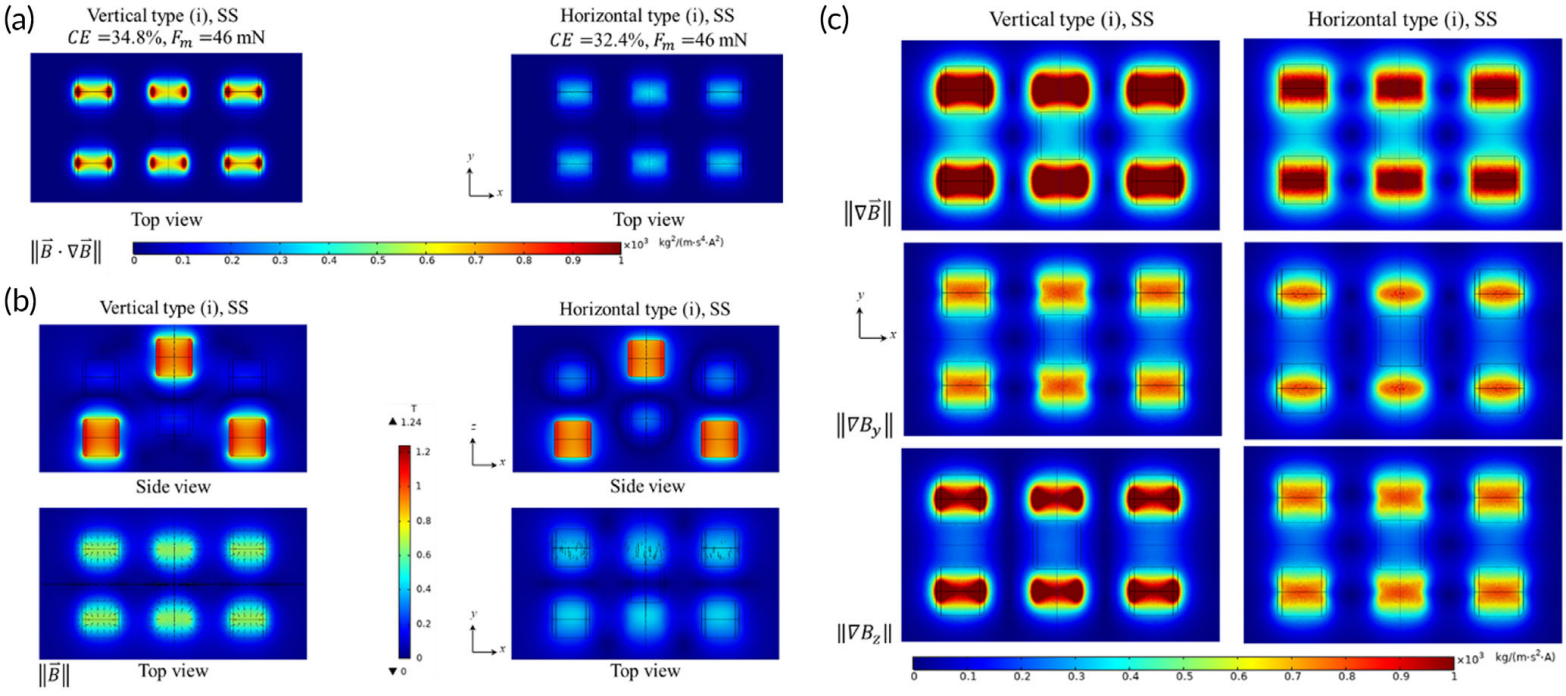
Magnetic field distributions for three‐stage filters of horizontal and vertical types (i) with the same connection types. (a) The vector magnitude distribution of the vector, $\overset{⃑}{B}\cdot \nabla \overset{⃑}{B}$. (b) The vector magnitude distribution of magnetic flux density, $\overset{⃑}{B}$. (c) The second‐order tensor magnitude distribution of the magnetic flux density gradient, $\nabla \overset{⃑}{B}$, with the components on the x‐y plane (top view); the vector magnitude distributions of the y‐component magnetic flux density gradient, $\nabla B_{y}$, and the z‐component magnetic flux density gradient, $\nabla B_{z}$.

### 3.5 Characteristic CE curves via dimensionless numbers

The CE is determined by the five independent dimensionless numbers as detailed in Section 2:^39^, ^40^

(3) $$\mathrm{CE}=\Phi \left(\mathrm{MH},\mathrm{Ar},\mathrm{As},N,m\right)$$

where MH is the ratio of the magnetophoretic force to hydrodynamic drag, Ar is the magnet to vessel cross‐sectional area ratio, As is the magnet aspect ratio, and m is the number of stages. Herein, the number of magnets, N, can be further subdivided to $N_{s}$ and $N_{p}$, representing the numbers of magnets deployed in series and parallel, respectively. $N_{s}$ and m are related to the magnet aspect ratio, As, proportional to the magnet length, $L_{M}$, while $N_{p}$ is more related to the cross‐sectional area ratio, Ar, directly proportional to the particle CE.^39^ In this study, $N_{s}$ remains a constant of 1 for any multi‐stage filters, while $N_{p}$ is 3 for single‐stage filters (m =1) and becomes 6 for multi‐stage filters with more than two stages (m≥2) since the additional stages are connected in the staggered way. Thus, the reduced functional relationship (3) can be rearranged as follows: $\mathrm{CE}/\mathrm{Ar}=\tilde{\Phi }\left(\mathrm{MH},\mathrm{As}\right)$ for single magnet filters (N=1, m=1), $\mathrm{CE}/\mathrm{Ar}=\tilde{\Phi }\left(\mathrm{MH},\mathrm{As},N_{s}\right)$ for series filters ($N_{p}=1$, m=1), $\mathrm{CE}/\left(N_{p}\mathrm{Ar}\right)=\tilde{\Phi }\left(\mathrm{MH},\mathrm{As}\right)$ for parallel (single‐stage) filters ($N_{s}=1$, m=1), and $\mathrm{CE}/\left(2N_{p}\mathrm{Ar}\right)=\tilde{\Phi }\left(\mathrm{MH},\mathrm{As},m\right)$ for multi‐stage filters with more than two stages ($N_{s}=1$, m≥2).

Figure 5a shows the CE for single, series, and parallel filters against MH for the fixed values of As and Ar with m=1. The simulation data, merged in x‐direction onto three curves (Figure S6b), were merged in y‐direction using $\mathrm{CE}/\left(\;N_{p}\mathrm{Ar}\right)$ across filter types: parallel ($R^{2}=0.987$), series ($R^{2}=0.967$), and single magnet ($R^{2}=0.936$) filters, respectively. These linear relationships suggest a suitable logarithmic equation, as follows:

(4) $$\mathrm{CE}/\left(\;N_{p}\mathrm{Ar}\right)=k{\left(\mathrm{MH}\right)}^{n}$$

where k is an empirical intercept determined from the data, and n is an empirical slope which is a function of $mN_{s}\mathrm{As}$. For parallel and single magnet filters, $mN_{s}\mathrm{As}$ remained the same at 1, indicating an identical empirical slope, n, of 0.55, while $mN_{s}\mathrm{As}$ was 3 for the series filters (n=0.53). For filters of identical size and magnet number, parallel deployment exhibited greater CE than series deployment due to the threefold higher $N_{p}\mathrm{Ar}$ value.

**FIGURE 5:**
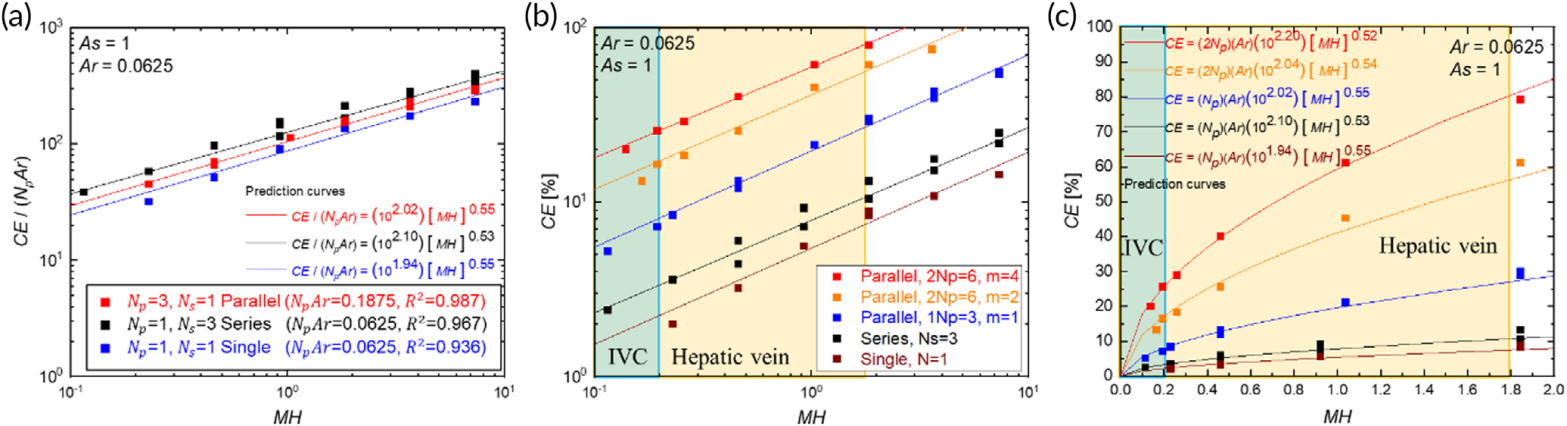
Particle capture efficiency against various values of the magnetophoretic to hydrodynamic force ratio (MH) for fixed values of the magnet aspect ratio (As) of 1 and the magnet to vessel area ratio (Ar) of 0.0625 with the prediction curves. (a) $\mathrm{CE}/\left(N_{p}\mathrm{Ar}\right)$ against MH where the red, black, and blue curves correspond to single magnet ($R^{2}=0.936$), series ($R^{2}=0.967$), and parallel ($R^{2}=0.987$) filters, respectively. (b) and (c) CE against MH where the brown, black, blue, orange, and red curves correspond to single magnet ($R^{2}=0.936$), series ($R^{2}=0.967$), parallel ($R^{2}=0.987$), two‐stage ($R^{2}=0.850$), and four‐stage ($R^{2}=0.920$) filters, respectively. The light blue and yellow boxes depict ranges of the inferior vena cava (IVC) and hepatic vein. (b) Data plot on a logarithmic graph. (c) Data plot on a linear graph.

Figure 5b,c depict an increase in CE with MH, illustrating prediction curves derived from a nonlinear regression curve‐fitting technique (4) within the regimes of the IVC and hepatic vein. The practical ranges of MH for clinical use are up to 0.2 and 1.8 for the IVC and hepatic vein, respectively. For fixed values of As and Ar, the performance of each filter, with magnets magnetized across the magnet's diameter, was summarized from single magnet to multi‐stage filters. The values of $mN_{s}\mathrm{As}$ were 2 for two‐stage filters (n=0.54) and 4 for four‐stage filters (n=0.52). The empirical slope, n, decreased (0.55, 0.54, 0.53, and 0.52) with the increase in the value of $mN_{s}\mathrm{As}$ (1–4, and), corresponding to single magnet and parallel (single‐stage); two‐stage; series and three‐stage; and four‐stage filters, respectively. Lower n values are advantageous for MH<1 to achieve higher CE, whereas higher n values are advantageous for MH>1. This trend indicates that an extended length of a device along the vessel (increased $mN_{s}\mathrm{As}$) increases the CE at low MH values (MH<1), but the rate of increase in the CE decreases. The CE is more sensitive to the coefficient of MH than n (4). The values of $N_{p}\mathrm{Ar}$ for single magnet and series filters were three times smaller than those for parallel (single‐stage) filters and six times smaller than those for multi‐stage filters (m≥2). The prediction curves of the CE from two‐stage to four‐stage filters were gradually increased, while the change of the increase in the CE between the single‐stage and two‐stage filters was relatively significant. Since the connected 2nd stage with the staggered way increased the number of magnets facing the blood flow, $N_{p}\mathrm{Ar}$ became two times higher ($2N_{p}\mathrm{Ar}$), explaining the significant increase in the CE between the single‐stage and two‐stage filters. The CE for each filter is most sensitive to $N_{p}\mathrm{Ar}$ among the independent dimensionless parameters, showing a linear dependence on $N_{p}\mathrm{Ar}$, while an approximately square root dependence on MH. These findings emphasize the crucial role of the distance between the filter and the particle in attracting particles towards magnets, which relates to $N_{p}\mathrm{Ar}$.^39^ The magnetic filter performance in the IVC presents challenges due to considerably faster flow, approximately 6–9 times greater than that of the hepatic vein, and a vessel diameter at least 1.5 times larger than that of the hepatic vein. These factors result in higher hydrodynamic drag and longer distances between the magnet and particles. For instance, at an MH of 0.2, particle CEs in the IVC were approximately 8% for single‐stage filters, 17% for two‐stage filters, 22% for three‐stage filters, and 26% for four‐stage filters. In contrast, at an MH of 1.8, particle CEs in the hepatic vein reached approximately 30% for single‐stage filters, 55% for two‐stage filters, 70% for three‐stage filters, and 80% for four‐stage filters. These findings suggest the feasibility of using multi‐stage filters in the hepatic vein to capture the excessive drugs. Utilizing characteristic curves with empirical values of k and n determined by magnetophoretic scaling laws can aid in the initial design of the magnetic filtration devices and prediction of their performance, despite inherent differences leading to variations among filter types.

## 4 DISCUSSION

### 4.1 Dimensionless scaling analysis

Particle CE in endovascular magnetic filtration is governed by the balance between magnetophoretic and hydrodynamic forces rather than by particle size alone. Magnetophoretic force scales with particle volume and magnetic field gradients ($F_{\mathrm{MP}}\sim d_{p}^{3}\nabla B^{2}$) (Equation S8), whereas viscous drag scales linearly with particle diameter ($F_{D}\sim d_{p}$) (Equation S4). As a result, the ratio $F_{\mathrm{MP}}/F_{D}$ scales as $d_{p}^{2}$. The characteristic CE curves show that CE follows a power‐law relationship with the magnetophoretic‐hydrodynamic parameter MH, that is, $\mathrm{CE}\sim MH^{n}$, where the emprically fitted exponent, n, ranged from 0.52 to 0.55. This empirical scaling implies an approximately linear dependence of CE on particle diameter ($\mathrm{CE}\sim d_{p}^{\left(2n\right)}\approx d_{p}$). The slight deviation of n from 0.5 reflects geometric and flow‐field effects captured in the simulations and experiments. Importantly, these characteristic CE curves enable scaling‐based extrapolation of capture performance to smaller particles without reliance on specific dimensions.

### 4.2 Comparison to in vitro experiments

Experimental data validated the empirical CE curves derived from dimensionless parameters based on the dimensionless scaling law of magnetophoretic systems for particle transport.^22^, ^23^, ^39^ For magnets deployed in series, simulations evaluated four models with both axially and diametrically magnetized magnets placed in alternating and unidirectional configurations (Table 1). The filters with diametrically magnetized magnets captured up to 1.9 times more particles than those with axially magnetized ones, consistent with experimental observations.^22^, ^23^ Minor discrepancies are likely caused by the particle aggregation, which was unaccounted for in numerical models. In experiments, particle aggregates near magnets increased effective particle volume, thus enhancing CE through stronger magnetophoretic force (Equation S8). Additional disparities may be explained by the difference between the particle sizes used in experiments and those reported by the manufacturer. The agreement between experimental and numerical results could be improved by incorporating particle clusters, wall effects, and Brownian motion of submicron particles into the governing equation (Equation S1). Additional details on the agreement between simulations and experiments were provided in our previous study.^39^ In summary, the agreement with experimental data validates our computational approach and demonstrates the potential of magnetic filters for in vivo capture of chemotherapeutic drugs.

### 4.3 Limitations and future work

This study simulated endovascular magnetic filters under simplified flow conditions and vessel geometries to focus on the dominant magnetophoretic capture physics. Although hepatic venous flow is pulsatile, and vessels are compliant, particle capture is governed primarily by the local balance between hydrodynamic and magnetophoretic forces. Under pulsatile conditions, flow unsteadiness introduces temporal fluctuations in MH through velocity variations, but does not alter the governing dimensionless force balance. In this study, blood flow in hepatic veins and IVC was assumed steady and the vessel wall was modeled as rigid.^29^ These assumptions may affect absolute capture values but are not expected to alter the scaling trends reported in this study. Blood flow was modeled as Newtonian continuum, as non‐Newtonian effects in vessels with diameters above 1 mm are likely to be negligible.^44^ Red blood cells and platelets were not explicitly modeled, allowing the analysis to focus on the dominant balance between hydrodynamic and magnetophoretic forces governing particle capture. While cellular components may influence local transport, margination, and particle‐cell interactions in vivo, these effects are secondary to the scaling‐based capture physics considered here. Future studies should investigate how blood cells and multiphase interactions may interfere with the capture of drug‐loaded magnetic particles. Additional clinical considerations related to blood flow are provided in Supporting Information S5.

Magnetic particle aggregation and interactions with blood cells represent important considerations for intravascular magnetic filtration. Aggregation can alter the effective hydrodynamic size and magnetophoretic response of particles, while red blood cells and platelets may promote hetero‐aggregation or intercept particles in vivo. In the present study, magnetic particles were assumed to be uniformly distributed at the inlet and remain uniformly dispersed to isolate the scaling‐based capture physics, with no interaction between particles.^45^ Several studies have proposed strategies to enhance magnetic particle stability and dispersion, including surface functionalization and encapsulation within stabilized carrier droplets, which can mitigate aggregation under physiological conditions.^46^ Incorporating such dispersion strategies, together with multiphase blood–particle interactions, will be an important direction for future experimental and computational studies to further improve capture efficiency and translational robustness. Moreover, in vitro studies cannot replicate all the physiological components of blood. In particular, hemocompatibility and thrombosis associated with platelets and coagulation factors in deployed filters represent important but distinct considerations for clinical translation. Near the magnets, magnetophoretic forces substantially exceed cell‐induced hydrodynamic perturbations, justifying the emphasis on particle‐scale transport physics in the present model. During IAC, the filter is deployed only for the duration of drug infusion and is subsequently removed, thereby reducing the risk of thrombus formation. Moreover, anticoagulant and antiplatelet medications are typically administered in clinical settings, further mitigating thrombogenic risk. Beyond pharmacological anticoagulation, the biocompatibility of endovascular magnetic filters can be improved through appropriate material selection and surface modification. Clinically established intravascular materials and surface treatments, such as hydrophilic or antithrombogenic coatings, have been shown to reduce platelet adhesion and thrombus formation. While this study focuses on magnetophoretic capture physics and scaling behavior, comprehensive evaluation of surface modification, material selection, and long‐term hemocompatibility will be addressed in future device development and in vivo studies.

Permanent magnets generate steady magnetic fields that penetrate biological tissues with minimal attenuation and without energy consumption. They can be designed with varied shapes and structural and magnetic properties, making them suitable for diverse biomedical applications. However, several design considerations must be taken into account. Particle capture depends on the number of magnets, their cumulative surface area, and magnet size; however, oversized magnets may increase a pressure drop across the device, thus impeding venous flow. Particle size is also a critical factor influencing CE. Rather than assuming a specific fixed size, particle diameters ranging from 0.5 to 2 μm were systematically examined to identify optimal and clinically admissible regimes using magnetophoretic scaling principles, although sub‐200 nm SPIONs are often emphasized in systemic magnetic drug delivery. Larger particles can be easily manipulated by magnetophoretic forces (Equation S8), while they must remain smaller than 2 μm to avoid capillary occlusion.^26^ Particle aggregation near the magnets could transiently exceed this size limit, but aggregates immediately disperse once they move outside the magnetic field.^39^ Accordingly, the present framework is intended to provide generalizable design rules across nano‐to‐micro‐scale magnetic carriers. The major challenge is to generate sufficiently strong magnetophoretic forces for capturing submicron particles. Thus, all relevant parameters in this study are optimized to maximize CE. Further, the structural design of multi‐stage filters must prevent magnets from collapsing onto each other within the vessel, which requires evaluation of magnetic interactions between magnets. Various architected device configurations for alternative drug capture mechanisms were proposed by Maani et al.^19^, ^24^ The proposed design of a perforated, twisted honeycomb‐shaped filter could offer advantages for structural safety as well as facilitating flow mixing, and will be modeled in future studies using magnetophoretic scaling arguments.

Clinical translation will require in vivo validation using large animal models providing physiological flow conditions and vessel compliance. Integration with delivery systems and thorough evaluation of biocompatibility, thrombogenicity, and magnetic safety are essential next steps. Once successfully validated in animal models, magnetic filtration could advance to early‐phase clinical testing during IAC procedures, with the potential to improve therapeutic outcomes while minimizing systemic toxicity.

## 5 CONCLUSION

This study demonstrated the potential of catheter‐based endovascular magnetic filters with permanent cylindrical magnets for capturing excess chemotherapeutic agents from the bloodstream. Multi‐physics simulations, coupling hemodynamic and magnetic field characteristics, were used to evaluate magnetic particle transport across alternative device configurations. The interplay between magnetophoretic and hemodynamic forces was found to play a dominant role in particle capture. The numerical results were found to align well with prior in vitro experiments.^22^, ^23^, ^39^ To optimize filter design for maximum CE and assess interactions between the magnets, we analyzed magnetic and magnetophoretic forces using the Maxwell magnetic stress tensor and magnetic field gradients. Multi‐stage filters with three diametrically magnetized cylindrical magnets per stage, achieved higher CE while mitigating interactions between the magnets. Leveraging magnetophoretic scaling laws,^39^ we derived predictive performance curves, estimating up to 80% particle capture efficiency for four‐stage filters deployed in the hepatic vein. These results establish a predictive modeling framework for magnetic filter design, enabling quantitative evaluation of device performance while reducing the need for animal testing. This work advances the development of clinically viable magnetic filters that can safely increase chemotherapeutic dosage, thereby shortening treatment duration and improving patient outcomes.

## AUTHOR CONTRIBUTIONS

**Won‐Seok Heo:** Conceptualization; methodology; software; investigation; validation; formal analysis; data curation; visualization; resources; writing – review and editing; writing – original draft. **Steven W. Hetts:** Conceptualization; resources; funding acquisition. **Vitaliy L. Rayz:** Writing – review and editing; supervision.

## FUNDING INFORMATION

This work was supported, in part, by NIH R01CA194533 (Hetts, PI).

## CONFLICT OF INTEREST STATEMENT

The authors declare no conflicts of interest.

## Supporting information


**Table S1.** Variable summarization.
S1. Theoretical backgrounds.
S2. Numerical approach.
**Figure S1.** Contour plots of the Maxwell magnetic stress tensor norm for single‐ and three‐stage filtration devices over the magnet and force probe surfaces.
S3. Magnetic forces exerted on magnets for parallel (single‐stage) filters.
**Figure S2.** Magnitude and direction of the magnetic forces for circumferential types (i) and (ii).
**Figure S3.** Magnitude and direction of the magnetic forces for horizontal types (i), (ii), and (iii).
**Figure S4.** Magnitude and direction of the magnetic forces for radial types (i) and (ii).
**Figure S5.** Magnitude and direction of the magnetic forces for vertical types (i), (ii), and (iii).
S4. Relative importance of the dimensionless numbers.
**Figure S6.** Particle capture efficiency (*CE*) distributions for fixed values of the inertial to hydrodynamic force ratio (IH) with magnet aspect ratio (As) of 1 and magnet to pipe area ratio (Ar) of 0.0625. The solid lines with different colors correspond to the types of filters: Parallel, Series, and Single magnet. The different symbols correspond to the constant values of IH. (a) CE against various values of the fluid magnetic to particle kinetic energy density ratio (${\mathrm{ER}}_{\mathrm{MK}}$). (b) CE against various values of the magnetophoretic to hydrodynamic force ratio (MH).
S5. Clinical considerations in blood flow.

## Data Availability

The data that support the findings of this study are available from the corresponding author upon reasonable request.
