# Supplementary material for: Endovascular magnetic filters for removing excess chemotherapeutic drugs from blood flow using dimensionless scaling laws
Source: Bioeng Transl Med. 2026 Oct 7;11(6):e70151. doi: 10.1002/btm2.70151 (PMC13646516; doi:10.1002/btm2.70151)
Supplement: Supplementary file 1 [file BTM2-11-e70151-s001.doc]

# **Supporting Information**

Endovascular magnetic filters for removing excess chemotherapeutic drugs from blood flow using dimensionless scaling laws

Won-Seok Heo^a,*,1^, PhD, Steven W. Hetts^b^, MD, and Vitaliy L. Rayz^a,c^, PhD

^a^ School of Mechanical Engineering, Purdue University, West Lafayette, IN 47907, USA

^b^ Department of Radiology and Biomedical Imaging, University of California, San Francisco, CA 94143, USA

^c^ Weldon School of Biomedical Engineering, Purdue University, West Lafayette, IN 47907, USA

^*^ Corresponding author. E-mail address: wheo@mgh.harvard.edu (W. -S. Heo).

^1^ Present address: Koch Institute for Integrative Cancer Research, Massachusetts Institute of Technology, Cambridge, MA 02139, USA

Table S1. Variable summarization

| Symbol | Meaning | Symbol | Meaning |
| --- | --- | --- | --- |
|  | Particle capture efficiency |  | Magnetic flux density |
|  | Number of magnets |  | Magnetic permeability of particle |
|  | Number of stages |  | Magnetic permeability of fluid |
|  | Length of magnet |  | Magnetic permeability of free space |
|  | Diameter of magnet |  | Relative magnetic permeability |
|  | Diameter of fluid vessel |  | Magnetic Clausius-Mossotti factor |
|  | Diameter of particle |  | Magnetophoretic force to drag ratio |
|  | Fluid velocity relative to particle |  | Particle inertia to drag ratio |
|  | Fluid dynamic viscosity |  | Magnet to vessel area ratio |
|  | Density of particle |  | Magnet aspect ratio |
|  | Density of fluid |  | Reynolds number |

# **S1. Theoretical backgrounds**

### **S1.1. Magnetic particle transport through flow and magnetic fields**

Magnetic particles in a flow field experience hydrodynamic drag and magnetophoretic force due to a magnetic field. Their transport can be described by Newton’s second law of motion:

|  |  | (S1) |
| --- | --- | --- |

where and are the mass and velocity of the particle, respectively.,, and denote the magnetophoretic, hydrodynamic, and gravitational forces. Herein, gravitational force is negligible in the human body since the ultra-small particles in the vessel are almost free from the gravitational influence. Particle inertia can also be ignored as the particle reaches terminal velocity almost instantaneously.^39^ Hence, the particle motion is determined by both flow and magnetic fields.

In this study, blood flow in the hepatic vein is assumed to be a steady and fully developed laminar flow of a Newtonian and incompressible fluid in a rigid blood vessel. The governing equations describing the blood flow are given by:

|  |  | (S2) |
| --- | --- | --- |

|  |  | (S3) |
| --- | --- | --- |

where,,, and are the density, velocity, pressure, and dynamic viscosity of blood, respectively. The vessel Reynolds number for blood flow in the hepatic vein () is approximately 150, indicating laminar flow, while the particle Reynolds number () is less than 0.123, indicating Stokes flow. It is assumed that the motion of ultra-small particles does not impact the blood flow field. The Stokes drag for spherical particles in blood vessels can be expressed as:

|  |  | (S4) |
| --- | --- | --- |

where is the diameter of the particle.

Magnetostatic fields generated by the magnets constructing the filters can be described by Maxwell’s equations in terms of magnetostatic scalar,, and magnetic vector potentials, with a constitutive relation:

|  |  | (S5) |
| --- | --- | --- |

|  |  | (S6) |
| --- | --- | --- |

|  |  | (S7) |
| --- | --- | --- |

where is the magnetic field strength, is the magnetic flux density, N/A^2^ is the magnetic permeability of free space, and is the magnetization of the material.

Magnetic fields are assumed continuous, indicating that the tangential component of and the normal component of are respectively identical across all boundaries including interface between inside and outside the particle, based on the curl-free condition on and divergence-free condition on.^32, 39^ Through these boundary conditions and Laplace’s equation,, the magnetic field strength inside the particle can be obtained.^39, 40^

The motion of magnetic particles driven by the magnetic field is assumed to have no interparticle effects and no effect on the magnetic field.^47^ The detailed derivation of magnetic particle magnetization is provided in the previous study.^39^ The magnetophoretic force acting on a magnetic spherical particle suspended in an electrically non-conducting fluid is given by:^32, 39, 47, 48^

|  |  | (S8) |
| --- | --- | --- |

where is the particle volume, is the magnetic permeability of the fluid, and is the magnetic Clausius-Mossotti factor for dipole, defined as.^49^ Here, is the magnetic susceptibility difference between the particle and fluid and is the relative magnetic permeability of the fluid. The sign of magnetophoretic force is determined by the sign of the Clausius-Mossotti factor: positive magnetophoresis () results in the factor greater than 0, and negative magnetophoresis () results in the factor less than 0.

### **S1.2. Magnetic interactions between magnets**

To prevent the magnets from colliding inside the blood vessel, we investigated the magnitude and direction of the magnetic force exerted on the neighboring magnets using both theoretical and computational models.

From Cauchy’s momentum equation in continuum mechanics, the electromagnetic force density,, can be expressed as the divergence of the Maxwell electromagnetic surface stress tensor,.

|  |  | (S9) |
| --- | --- | --- |

By taking a volume integral of the divergence of the magnetic surface stress tensor,, over a closed surface and applying the divergence theorem, the magnetic force,, between the magnets can be derived as:

|  |  | (S10) |
| --- | --- | --- |

where is the normal unit vector, is the boundary of the domain, and the subscripts 1 and 2 refer to the regions inside and outside a body, respectively. This equation provides the magnetic force exerted on the exterior surface of the body. In magnetostatics, the Maxwell magnetic surface stress tensor,, can be expressed directly based on the magnetic flux density,, and magnetic field strength,, at the boundary as below.

|  |  | (S11) |
| --- | --- | --- |

The magnitude and direction of the magnetic forces can be numerically obtained using this equation.

### **S1.3. Magnetophoretic force acting on the particles**

The magnetophoretic force is proportional to the product of the magnetic flux density and its spatial derivative. The magnetic flux density gradient,, a second-order tensor, can be expressed with respect to the,, and directions as below.

|  |  | (S12) |
| --- | --- | --- |

In the irrotational magnetic field, the field of is simply identical to.

|  |  | (S13) |
| --- | --- | --- |
|  |  | (S14) |

Also, the magnitude of the second-order tensor can be obtained using the tensor properties where. Thus, the tensor norm can be calculated as the square root of the double contraction of two same tensors. By qualitatively and quantitatively comparing the magnitudes of the first-order and second-order tensors of the magnetic fields, the particle capture efficiency () can be estimated without solving the particle motion equation, leading to a reduction of computation time.

In this work, the magnets are diametrically magnetized, meaning the magnetization is normal to the magnet’s axis, corresponding to the-direction in Figure 1c-d. Therefore, the-component of the magnetic flux density () and the spatial derivative with respect to () are negligible compared to the other components. Under these assumptions, the magnetic flux density gradient () can be reduced as a two-dimensional matrix as below.

|  |  | (S15) |
| --- | --- | --- |

Similarly, the-component of the vector can be removed, resulting in only and components as below.

|  |  | (S16) |
| --- | --- | --- |
|  |  | (S17) |

This two-dimensional analysis dominates the assessment of the magnetophoretic force for predicting the particle.

# **S2. Numerical approach**

### **S2.1. Magnitude of the magnetic stress tensor**

When computing the forces using the Maxwell surface stress tensor (Equation S10), accurately calculating the flux integrals for the magnetic flux density, , and magnetic field, , is crucial. As the fields near the magnet poles are highly concentrated, so is the stress tensor at the sharp corners. Consequently, the quantities entering the boundary integral become concentrated in a few mesh elements, with nearly zero values elsewhere. This concentration can cause numerical inaccuracies when computing magnetic forces on magnets with sharp edges.

If there is enough space around the body of interest, one alternative to address this issue is to introduce an auxiliary surface that encloses the magnet while maintaining a certain distance, referred to in this model as a force probe surface. The singular behavior occurring near the poles diminishes at a distance, while the stress tensor behaves more nicely at a certain distance from the magnet. Using fillets, advanced meshing, and exterior force-probe surfaces improves the accuracy of the resulting integral. To visualize the magnetic forces between magnets, the distributions of the Maxwell magnetic stress tensor norm on the surfaces of the magnet and force probe for multi-stage filtration devices of vertical type (i) with the same connection types are depicted in Figure S1. The closed force probe surface is enlarged to avoid interference with other magnets, resulting in smaller stress tensor magnitudes on the force probe surface compared to the magnet surface due to the extended surface area. However, the magnetic force remains the same as below.

|  |  | (S18) |
| --- | --- | --- |

To achieve an accurate stress tensor near the magnet edge, the stress tensor magnitude is represented over the force probe surface. The force probe surface is designed with an ellipsoid-like shape to reduce sharp corners, resulting in a larger surface area, smaller Maxwell stress tensor, and identical magnetic force. The stress tensor norm distribution (Equation S11) allows for the comparison of the magnitudes of the magnetic force.

Figure S1. Contour plots of the Maxwell magnetic stress tensor norm for single- and three-stage filtration devices over the magnet and force probe surfaces.

### **S2.2. Spatial derivatives of the magnetic field**

The COMSOL software provides numerical methods for calculating magnetic fields using the magnetic potential equations of (Equation S5 and S6). The curl element (edge or vector element) is widely used in the finite element method (FEM) to solve the first-order spatial derivatives of the magnetic scalar and vector potentials. However, since a second-order spatial derivative is not defined on the curl element in FEM, the gradients of and cannot be obtained from the curl element directly. Instead, the Lagrange elements can be used to compute the magnetic potential equations, allowing for the computation of second-order spatial derivatives. Through this numerical method, each component of the magnetic flux density can be mapped to an auxiliary field by adding an extra equation with three unknown variables (one scalar field for each component of ). These new variables use the second-order Lagrange element. Since both first-order and second-order spatial derivatives are defined on the Lagrange element, it becomes possible to obtain the spatial gradients of the and . The first step is to map the magnetic flux density onto the Lagrange elements. To obtain the appropriate expression for mapping the field components, the calculated field components are mapped to an auxiliary vector field in a partial differential equation (PDE). Consequently, when solving the PDE from the computed results of the magnetic fields, the components of the magnetic flux density are directly mapped onto the three scalar fields of the auxiliary vector field. This process enables the numerical computation of the three components of the magnetic flux density gradient: , , and (Equation S12).

# **S3. Magnetic forces exerted on magnets for parallel (single-stage) filters**

Figure S2 ~ S5 illustrate the distribution of magnetic forces within the filters. Red arrows denote the magnetization directions of individual magnets, blue arrows represent the direction of the strongest force among three magnets with their relative magnitudes indicated, and dark grey arrows depict the force directions for the remaining magnets. As shown in Figure S2, magnets of circumferential type (i) experience attractive forces of equal magnitude directed towards the vessel’s center, while magnets of type (ii) experience repulsive forces with the top magnet experiencing the strongest force. The bottom two magnets attract each other with a 60-degree downward in the simulation coordinate system (Figure 1c). The force ratios between magnets for types (i) and (ii) are 1:1:1 and 1:1:, respectively. As shown in Figure S3, Horizontal types (i) and (ii) exhibit similar force patterns. Type (i) features attractive forces directed towards the center, while type (ii) experiences repulsive forces with different angles of 11 and 4 degrees, respectively. Type (iii) displays a more complex repulsive force pattern compared to types (i) and (ii). As shown in Figure S4, magnets of radial type (i) experience repulsive forces of equal magnitude, while type (ii) showed the strongest force directed towards the vessel’s center and the remaining two magnets experience repulsive forces directed outwards at a 60-degree angle. As shown in Figure S5, magnets of vertical type (i) experience attractive forces, while type (ii) experience repulsive forces with 14-degree outward direction. Type (iii) exhibits relatively complex force distribution, with each magnet experiencing a distinct force direction where the strongest force acts along the horizontal direction. For configurations with antisymmetric magnetic fields (circumferential and horizontal type (i)s and (ii)s), the tangential components of the magnetic field on the - plane are zero. Conversely, configurations with symmetric fields (radial and vertical type (i)s and (ii)s) lack normal components on the - plane with all non-zero components being tangential. Notably, both symmetric and antisymmetric magnetic fields result in symmetrical distributions of magnetic forces. Table 2 summarizes the maximum magnetic forces for all single-stage filter configurations, represented by the blue arrows in the figures. While the net magnetic forces for all models (Figure S2 ~ S5) are zero, horizontal and vertical type (iii)s exhibit non-zero net moments (Figure S3 and S5). Filters with non-zero moments are susceptible to rotational motion.

Figure S2. Magnitude and direction of the magnetic forces for circumferential types (i) and (ii).

Figure S3. Magnitude and direction of the magnetic forces for horizontal types (i), (ii), and (iii).

Figure S4. Magnitude and direction of the magnetic forces for radial types (i) and (ii).

Figure S5. Magnitude and direction of the magnetic forces for vertical types (i), (ii), and (iii).

# **S4. Relative importance of the dimensionless numbers**

The proposed magnetic filters were designed as a positive magnetophoretic model where superparamagnetic particles are suspended in blood. The values of the magnetic susceptibility of blood, , were experimentally reported as and for the oxygenated blood in an artery (diamagnetic) and deoxygenated blood in a vein (paramagnetic), respectively.^50^ Since the values of each susceptibility are negligibly small, the relative magnetic permeability of blood () can be assumed to be approximately 1 for most biological tissues, blood, and water. On the other hand, the relative magnetic permeability of superparamagnetic particles, , is approximately 4000, indicating the magnetic Clausius-Mossotti factor, , remains approximately 1. In addition, the particle depends on the vessel Reynolds number containing blood flow velocity. However, is no more linearly independent in (Equation 4) since and include such that and , where is the ratio of the fluid magnetic energy density to particle kinetic energy density.^39^ Lastly, the functional relationship (Equation 4) can be reformulated by replacing with , given that . Figure S6 illustrates the particle dependence on the two dimensionless numbers of and while keeping all other dimensionless numbers fixed. As shown in Figure S6a, displays a linear relationship with on a logarithmic scale for different constant values, irrespective of filter types represented by different colors. For a fixed , increased with . Conversely, for a fixed , depended on the filter type. The filtration device with three magnets deployed in parallel (red) exhibited higher than the series deployment filer (black) and the single magnet device (blue). For the plot in Figure S6b, all data expressed by merged in -axis with identical , , and , regardless of the variation in . In fact, the values are almost zero since particle inertia is negligible compared to the Stoke’s drag and magnetophoretic force.^39^

Figure S6. Particle capture efficiency () distributions for fixed values of the inertial to hydrodynamic force ratio () with magnet aspect ratio () of 1 and magnet to pipe area ratio () of 0.0625. The solid lines with different colors correspond to the types of filters: Parallel, Series, and Single magnet. The different symbols correspond to the constant values of . (a) against various values of the fluid magnetic to particle kinetic energy density ratio (). (b) against various values of the magnetophoretic to hydrodynamic force ratio ().

# **S5. Clinical considerations in blood flow**

Blood is a complex suspension of erythrocytes, leukocytes, and platelets in plasma, making the flow complicated with slightly blunted velocity profiles. As blood exhibits shear-thinning behavior attributed to the resilience and deformability of red blood cells (RBCs), it behaves as a Newtonian fluid under high shear rates (> 100 s^-1^) or in vessels with diameters exceeding 1 mm, which is approximately 100 times the size of the RBC.^44^ The Fahraeus effect owing to the RBCs’ characteristics indicates that RBCs tend to align in the direction of flow along the center of the blood vessel, creating an RBC-free layer (RBC-FL) near the vessel wall with vessel diameter below 500 μm. This RBC alignment contributes to reduced apparent viscosity of blood, facilitating easier blood flow. In this environment, microparticles suspended in blood tend to be marginated towards the RBC-FL to avoid interaction with RBCs, while nano-sized spherical particles below 1 μm in a vessel with 1-cm diameter are well-mixed.^45^ In this study, magnetic particles were uniformly distributed at the vessel inlet, assuming non-interacting particles, although potential interference from blood cells with capturing drug-loaded magnetic particles needs to be further investigated in future studies.
